# Artificial Intelligence-Driven Modeling for Hydrogel Three-Dimensional Printing: Computational and Experimental Cases of Study

**DOI:** 10.3390/polym17010121

**Published:** 2025-01-06

**Authors:** Harbil Bediaga-Bañeres, Isabel Moreno-Benítez, Sonia Arrasate, Leyre Pérez-Álvarez, Amit K. Halder, M. Natalia D. S. Cordeiro, Humberto González-Díaz, José Luis Vilas-Vilela

**Affiliations:** 1 Department of Physical Chemistry, University of Basque Country UPV/EHU, 48940 Leioa, Spain; harbil.bediaga@ehu.eus (H.B.-B.); leyre.perez@ehu.eus (L.P.-Á.); 2 Department of Organic and Inorganic Chemistry, University of Basque Country UPV/EHU, 48940 Leioa, Spain; sonia.arrasate@ehu.eus (S.A.); humberto.gonzalezdiaz@ehu.eus (H.G.-D.); 3 BCMaterials, Basque Center for Materials, Applications and Nanostructures, UPV/EHU Science Park, 48940 Leioa, Spain; 4 LAQV-REQUIMTE, Department of Chemistry and Biochemistry, Faculty of Sciences, University of Porto, 4169-007 Porto, Portugal; amit.halder@fc.up.pt (A.K.H.); ncordeir@fc.up.pt (M.N.D.S.C.); 5 Dr. B. C. Roy College of Pharmacy and Allied Health Sciences, Durgapur 713206, India; 6 Basque Center for Biophysics, CSIC-UPV/EHU, 48940 Leioa, Spain; 7 IKERBASQUE, Basque Foundation for Science, 48011 Bilbao, Spain

**Keywords:** machine learning, artificial intelligence, database, hydrogel, modeling, bioprinting

## Abstract

Determining the values of various properties for new bio-inks for 3D printing is a very important task in the design of new materials. For this purpose, a large number of experimental works have been consulted, and a database with more than 1200 bioprinting tests has been created. These tests cover different combinations of conditions in terms of print pressure, temperature, and needle values, for example. These data are difficult to deal with in terms of determining combinations of conditions to optimize the tests and analyze new options. The best model demonstrated a specificity (Sp) of 88.4% and a sensitivity (Sn) of 86.2% in the training series while achieving an Sp of 85.9% and an Sn of 80.3% in the external validation series. This model utilizes operators based on perturbation theory to analyze the complexity of the data. For comparative purposes, neural networks have been used, and very similar results have been obtained. The developed tool could easily be applied to predict the properties of bioprinting assays in silico. These findings could significantly improve the efficiency and accuracy of predictive models in bioprinting without resorting to trial-and-error tests, thereby saving time and funds. Ultimately, this tool may help pave the way for advances in personalized medicine and tissue engineering.

## 1. Introduction

Three-dimensional bioprinting has revolutionized tissue engineering and regenerative medicine. This groundbreaking technique involves the manufacture of biologically functional structures using so-called bio-inks that contain cells, growth factors, or biomaterials. Hydrogels, which are three-dimensional cross-linked polymeric networks [1], represent one of the most common components of bio-inks, providing structural integrity to the printed tissues [2]. In fact, hydrogels owe their unique suitability as bio-ink carriers to their biocompatibility [3], water content, and the possibility of adjusting mechanical and biochemical properties. Examples of commonly used hydrogels in 3D printing are alginate [4], collagen [5], and hyaluronic acid [6], among natural materials, and synthetic polymers like polyethylene glycol [7].

In this context, printability, which is the ability to outline and maintain reproducible 3D scaffolds from bio-ink, is essential for the accurate fabrication of complex tissues and organs [8,9]. The factors that determine high-quality printability can be categorized into three groups. First are those related to the design of the scaffold, such as the size or thickness of the layer. Second are those related to the printing process, that is, pressure, temperature, or printing speed. Finally, the physical properties and rheological characteristics of the bio-ink are, obviously, determining factors for good printability [10].

Artificial intelligence (AI) and machine learning (ML) are fields that focus on the creation of algorithms that confer computers with capabilities of learning from data in order to improve their performance in specific tasks without explicit programming [11]. These technologies have been used in various fields of knowledge, such as medicine [12], pharmacology [13,14,15,16,17,18], speech recognition [19], agriculture [20], the petrochemical industry [21] computer vision [22], and the design and development of new materials. In fact, the convergence of AI and material science, which is a hot topic nowadays, represents a paradigm change in the development of new polymers, saving experimentation time, chemical consumables, and, consequently, funds. Despite the great potential of ML techniques, to the best of our knowledge, a limited number of works relating ML capabilities to the use of hydrogels as bio-inks in 3D printing techniques have been described. Among the first applications in this area, Elbadawi et al. [23] used ML algorithms to predict the printability of polymeric filaments in drug delivery by using fused deposition modeling. On the other hand, Nadernezhad and Groll [24] used the random forest algorithm to predict the printability of hyaluronic acid-based hydrogels, employing rheological properties.

There is no predefined minimum of data to train a predictive model, but the larger the dataset, the more refined the model and the better the analyses of the relationships among the variables. In the case that there is no existing database, information fusion (IF) methods can be applied to data from several studies or databases that may be integrated to create a more complete dataset [25]. This fusion aims to interactively consider all available information in order to obtain a wider and more detailed overview of the problem. Lastly, perturbation theory (PT) operators are mathematical tools that enable one to calculate the properties and behavior of a system beyond simple models [26]. IF, PT, and machine learning integration is an effective strategy to address the challenges of material design; it has gained successful applications in medicinal chemistry, proteomics, and nanotechnology, especially in handling a large volume of data with Big Data characteristics [14,16,17,18,25,26,27,28,29,30,31,32,33].

In this study, a versatile IFPTML model for predicting 3D-printed scaffold properties based on monomer structure is described. This application of AI/ML in 3D printing aims to enhance the optimization of crucial processes, materials, and printing parameters. These algorithms streamline optimization, enable real-time error detection, and reduce the number of iterative steps required for bio-ink formulation. To achieve these improvements, a database of experimental values documented in the literature was compiled. Various AI/ML-based models were trained to forecast upcoming bioprinting experiments and assist in the design of innovative monomers that ensure high printability of the corresponding hydrogels. All the trained models in this work are available in the following repository: https://github.com/hbediaga/hydrogel.git (accessed on 4 November 2024).

## 2. Materials and Methods

### 2.1. Computational Methods

#### 2.1.1. Database Creation

The models trained in this study are based on a database created from scratch by combining experimental data obtained by some members of our research group with data obtained from the literature (see Table 1) [34,35,36,37,38,39,40,41,42,43,44]. The bioprinting parameter data obtained consist of one or two hydrogels for each scaffold. The final database includes 1568 assays examining the bioprinting of 10 different hydrogels with a wide range of compositions and printing conditions. In these studies, 16 different properties were analyzed, including uniformity, porosity, printability, viscosity, width, and diameter, among others. These properties have been unified using methodologies based on IF. The information obtained from the literature has been grouped based on the conditions used for printing and the measured property of the bioprinted scaffold. To create a consolidated model, the results were categorized into two classes: good (1) and poor (0). The classification of a good or poor impression has been derived from the literature. These parameters have been defined in Table 2. Thus, if the parameter values in a given print were obtained within the limit values, it was considered good (1). Conversely, if the values went outside the predetermined range, it was considered poor (0). In this way the data could be analyzed comprehensively, enabling the development of a multi-property model. The result of each assay was expressed by one experimental parameter v_ij_ used to quantify the physicochemical properties of the ith hydrogel (m_i_) over the jth target. The values of v_ij_ depend on the structure of the hydrogel and on a series of boundary/experimental conditions that delimit the characteristics of the assay c_j_ = (c_0_, c_1_, c_2_, …c_n_). The first c_j_ was c_0_ = the measured property (porosity, uniformity, etc.). Other conditions are shown in Table 1. Considering this, in many cases, the compiled values v_ij_ are not exact numbers; classification techniques were used instead of regression methods, and values were discretized as follows: f(v_ij_)_obs_ = 1 when v_ij_ > cut-off.

#### 2.1.2. Descriptor Calculation

In addition to the experimental data previously described, the database was completed with the values of the molecular descriptors that serve as the foundation for converting categorical variables into numerical ones. To obtain the values of these molecular descriptors, the SMILES code [45] of the monomeric unit that forms the hydrogel polymer was employed. The Python [46] libraries Mordred [47] and alvaDesc [48] from OCHEM [49] were used to extract these molecular descriptor values. Both options are available free of charge. In the case in which the bio-ink was composed of two hydrogels, the values of the descriptors are added as a proportion of the concentration of each one.

**Table 2:** Bibliography used for the creation of the database and detailed summary of the selected compounds.

Author	HA ^a^	ChiMA ^b^	Gelatin	Alginate	MC ^c^	Agarose	NOOC ^d^	GelMA ^e^	GG ^f^	Chitosan	Ref.
Aguado et al.				x							[34]
Almeida et al.										x	[35]
Butler et al.						x	x				[36]
Chen et al.				x					x		[37]
Di Giuseppe et al.			x	x							[41]
Firipis et al.				x		x					[39]
Gao et al.			x	x							[50]
Jain et al.								x			[42]
Maíz-Fernandez et al.	x	x									[44]
Negrini et al.					x						[38]
Ouyang et al.			x	x							[43]
Soltan et al.			x	x							[51]

^a^ Hyaluronic acid; ^b^ Chitosan methacryloyl; ^c^ Methyl cellulose; ^d^ *N*,*O*-carboxymethyl chitosan; ^e^ Methacrylated gelatin; ^f^ Gellan gum.

#### 2.1.3. Outcome Classification

As previously mentioned, the database comprises measurements of 16 properties associated with the printability of hydrogels. Given the considerable number of properties intended for analysis, the results were classified into two categories. On the one hand, experiments with property values close to the desired value or the average of experiments (in cases in which no specific desired value was available) are designated as “1”. On the other hand, experiments with results that do not meet the desired criteria, whether above or below the established limits, are classified as “0”. To establish these thresholds, a search for a range that would allow experiments to be classified as “1” was conducted, ensuring a representative number of cases in both classifications and a balance between them. This process starts with the average or desired value, tallying the data in each group with varying deviations. The optimal classification was derived from the classification thresholds or cut-offs presented in Table 3.

#### 2.1.4. Model Generation Process

The PTML modeling technique is useful for seeking predictive models for complex datasets with multiple Big Data features. Scoring function values f(v_ij_)_calc_ for the ith hydrogel in the jth process conditions with multiple conditions of assay c_j_ = (c_0_, c_1_, c_2_, …c_n_) can be predicted. PT operators similar to Box–Jenkins Moving Average (MA) [52] operators ∆D_k_(c_j_) are used as input [53] for the kth descriptor. The development of linear IFPTML models holds immense promise in the field of 3D bioprinting. These models offer a structured and interpretable approach for predicting printability properties and classifying compounds. By integrating data from various sources, these models can effectively capture the relationships among monomer structures, printing parameters, and printability outcomes.

**Table 3:** Summary of results, including ranges of values (minimum, maximum, average) and distribution of rankings (n_0_ and n_1_) for each possible outcome.

Outcome (Unit)	Lim. Inf.	Avg	Lim. Supp.	n_0_	n_1_	Ref.
Uniformity, U	0.93	0.98	1.03	62	36	[44,50,51]
Pore factor, Pr	0.90	0.95	1.00	28	11	[8,39,42,43,51]
Integrity factor, I	0.30	0.61	0.70	16	13	[50,51]
Viscosity (cP)	1800.00	3054.86	15,000.00	10	4	[34,51]
Accuracy, Ac	82.82	87.18	91.54	12	3	[41]
Width (mm)	0.32	0.33	0.35	3	3	[41]
Parameter Optimzation Index, POI	40.00	57.04	65.00	3	3	[54]
Compr. Modulus (kPa)	35.00	38.13	42.00	3	3	[55]
Storage, G′ (Pa)	25.00	468.10	95.00	12	7	[34,36,50]
Loss moduli, G″ (Pa)	0.40	0.75	0.85	5	1	[36]
tan(G″/G′)	0.20	0.32	0.40	6	4	[50,51]
Swelling ratio, Sw	10.71	11.28	11.84	2	2	[37]
E (Pa)	100.00	830.99	2000.00	2	4	[50]
Diameter (mm)	100.00	735.44	772.21	18	30	[38]
Porosity (%)	78.00	77.35	85.00	1	1	[35]
Expansion (%)	8.00	10.18	25.00	628	632	[44]
Total				811	757	

IFPTML models allow researchers to understand the underlying factors influencing printability. This transparency is crucial for refining and optimizing the bioprinting process. Structural features of monomers with the most significant influence on printability can be detected, allowing for the proposal of specific modifications and design improvements.

The IFPTML model starts with the expected value of a printing property and adds the effect of different perturbations in the system. Consequently, the model has two types of input variables: the expected value function f(v_ij_)_expt_ and the PT operators D_k_(c_j_). The input variable f(v_ij_)_expt_ represents the expected value of the printing property for hydrogel in different combinations of printing conditions: c_j_ = (c_0_, c_1_, c_2_, … c_j_ … c_max_). The other PT operators are MA-calculated for one condition at a time. Therefore, PT operators can be calculated as follows: D_k_(c_j_) = D_ki_ − <D_k_(c_j_)>. The operators depend on the value of the molecular descriptor D_ki_ of type k used to quantify the structure of the ith monomer. The PT operators measure the deviation of D_ki_ from the expected value of <D_k_(c_j_)> (average value) of this descriptor for different sets of monomers c_j_. The output of the model f(v_ij_)_calc_ is a scoring function of the value v_ij_ of printing property of the ith monomer in the different combinations of the conditions of the assay c_j_. See Supplementary Materials file S1 for the database.

## 3. Experimental Methods

### 3.1. Bioprinting Conditions

Separate solutions of methacrylated chitosan (ChiMA, 1.5% (*w*/*w*)) dissolved in 0.5% (*v*/*v*) acetic acid and polyethylene glycol diacrylate (PEGDA, 20 mM) were prepared. Then, a solution of PEGDA was added to the CHIMA solution and stirred for 24 h. The photoinitiator lithium phenyl-2,4,6-trimethylbenzoylphosphinate (LAP, 0.1% (*w*/*w*)), previously dissolved in 300 μL of 0.5% (*v*/*v*) acetic acid solution, was added.

A bioprinter (INKREDIBLE + Cellink) (Cellink, Gothenburg, Sweeden) was used to print the hydrogel scaffolds, which were previously prepared and loaded into a cartridge (Adhesive Dispensing Ltd., Bletchely, UK). Hydrogel scaffolds were printed with a cell side length of 5 mm at a speed of 600 mm/min, with a nozzle inner diameter of 0.254 mm and a pressure of 21–27 kPa. Subsequently, the hydrogel grid was photopolymerized in situ with an ultraviolet LED light (405 nm, 19 mW/cm^2^) [44].

### 3.2. Image Caption and Analysis

The grid was analyzed with an optical magnifying glass (Nikon AZ100 Multizoom, Tokyo, Japan), and photos were taken at one and two magnifications. With the ImageJ 1.49 software, the impression of the hydrogel lines was analyzed. In particular, the expansion ratio and the uniformity factor were calculated [56].

## 4. Results and Discussion

### 4.1. Computational Model

Python (3.9.11) code has been developed to process the data and generate the models presented in this section. The best results obtained for each type of model are shown. These models can be used to score the properties of a new hydrogel in different combinations of assay conditions. Firstly, the reference function value must be substituted in the selected equation. It is noteworthy that these values change for different properties. Consequently, the model can predict different kinds of property parameters for a single hydrogel. Subsequently, the descriptors’ values for a new hydrogel must be input into the model. These values were determined using various calculation methods. In order to calculate these expected values of probability, we have to evaluate the following formula: p(f(v_ij_)_obs_ = 1)_expt_ = n(f(v_ij_) = 1)_obs/nj._ This ratio was computed by dividing the number of monomers, denoted as n(f(v_ij_) = 1)_obs_, that satisfy the specified ratio for condition c_j_ by the total number of compounds, n_j_, tested under the same condition, c_j_. The models presented are all based on the IFPTML process explained in the previous section.

#### 4.1.1. IFPTML Linear Model

**Figure 1:**
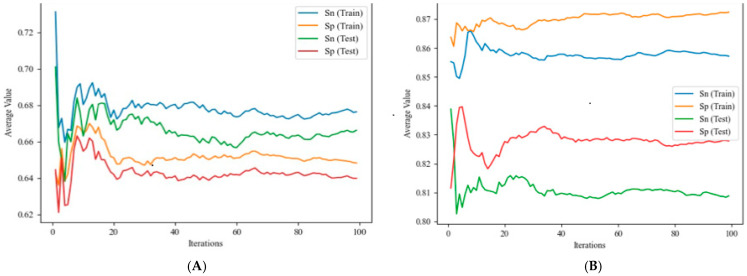
Comparison of sensitivity (Sn) and specificity (Sp) for the training and validation sets after bootstrapping with increasing training iterations. (**A**) Results for the IFPTML-LDA model; (**B**) results for the IFPTML-DTC model.

The linear discrimination analysis (LDA) model is a statistical method for finding a linear combination of attributes or characteristics to separate into groups. In the particular case of an LDA model, f(v_ij_)_calc_ is not in the range of 0–1 and is not a probability. The effectiveness of this type of model is based on the simplicity of its equations. However, it is important to note that the predictions obtained may not have a high degree of confidence. However, for a given value of f(v_ij_)_calc_, the LDA algorithm can calculate the respective values of posterior probabilities: p(f(v_ij_) = 1)_pred_ [57]. The model has two types of input variables: the expected value function f(v_ij_)_expt_ and the PT operators D_k_(c_j_). As mentioned above, the database has many input variables. In order to obtain a model with as few variables as possible, recursive feature elimination (RFE) selection was carried out [58]. In order to analyze the quality of the model, bootstrapping with 100 iterations was performed (see Figure 1A). With this method, taking the coefficients of the variables as a characteristic, less important sets were selected and eliminated from the model. This procedure was repeated until a model with 10 variables was obtained. Unfortunately, although this model usually gives good results, in this case, the selectivity and specificity parameters were not good (see Table 4).

After evaluating the results obtained with the IFPTML-LDA model, the possibility of improving class differentiation by refining the threshold definition functions was investigated.

#### 4.1.2. IFPTML Nonlinear Models

Finally, some nonlinear algorithms were investigated in order to achieve improvements in model performance compared to linear models. On the one hand, we trained decision tree classifier (DTC) and different artificial neural network (ANN) models. The IFPTML-DTC model utilizes the framework of a decision tree, employing a flowchart-like structure in which decisions are determined at each node based on specific attributes. Upon completion, each branch corresponds to a class, and each path, from the root to the final branch, defines a classification rule. In this case, as in the linear model, 100 iterations of the model have been analyzed to obtain the best result. The values of the model quality parameters over the iterations can be seen in Figure 1B, and the results of the parameters obtained for the best model can be seen in Table 4.

Although this model exhibits commendable performance in relation to the training set, the values for the statistical parameters of the validation set show a slight decrease. This difference in value was not observed in the case of the linear IFPTML-LDA model. As for the values of the parameters themselves, those obtained in the IFPTML-DTC model are much better, and it was mainly for this reason that this model was chosen as the best obtained and used to make the predictions. Nevertheless, all these models are available in the repository for use. The IFPTML-DTC model shown in Table 4 consists of 15 variables and a maximum of 40 leaf nodes.

**Figure 2:**
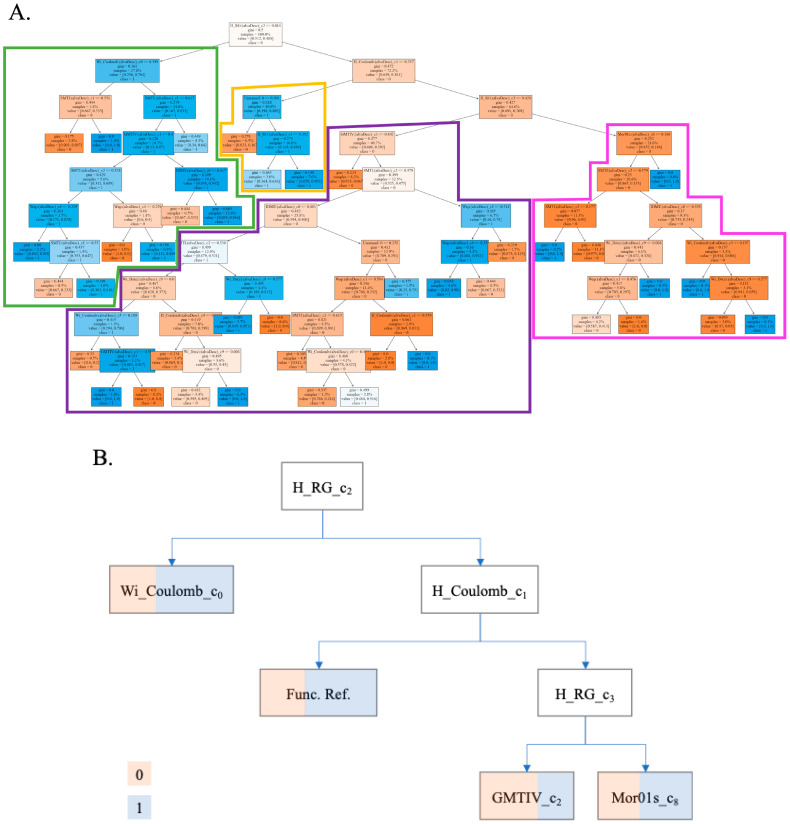
(**A**). Structure of the IFPTML-DTC model, showing all nodes and leaves with indicative classification colors. The branches highlight the different families differentiated in the model. (**B**). Simplified schematic of the main families identified, highlighting the key groupings obtained in the model.

The structure of the tree obtained can be seen in Figure 2A. By schematizing and splitting this tree for analysis, we can see four main families (Figure 2B). These families are obtained from the first three nodes of the tree. The first family (green) describes 27.8% of the data and arises directly from the main node of the model. The second (yellow) (10.6%), third (purple) (40.7%), and fourth (pink) (21.0%) families come from the same node.

**Figure 3:**
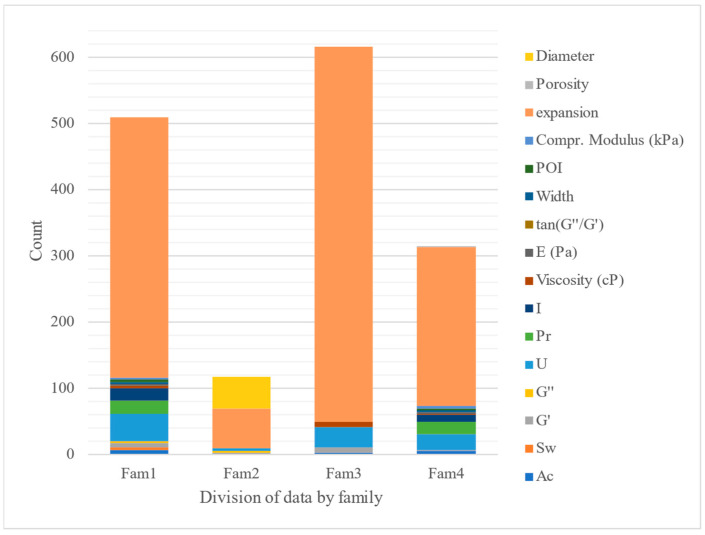
Distribution of the properties measured in each family, showing the variability and range of values observed within each identified group.

The first family differs from the rest because the hydrogels included in it have an input variable ∆HRG(c_2_) value greater than 0.014. This variable is equal to ∆HRG(c_2_) = HRG_i_ − <HRG(c_2_)> (see Table 5 for descriptions of each descriptor). As can be seen in Table 6, the first term of ∆HRG(c_2_) is the index HRG_i_ of the repetitive unit of the hydrogel. The value HRG_i_ is the Harary-like index from the reciprocal squared geometrical matrix. Then, HRG_i_ accounts for the number of interactions/contacts between non-bonded atoms. This means that a high HRG_i_ may indicate that the hydrogel repetitive unit is more compact or branched (more folded over itself) [59]. On the other hand, the second term <HRG(c_2_)> is the average value of HRG_i_ (expected value) for all hydrogels measured under the same condition c_2_ = extrusion speed. Consequently, the hydrogels included in this family are those with deviations greater than 0.014 in the structural branching of the unit, with respect to those measured at the same extrusion speed and output property. This implies that to design new hydrogels, we should fine-tune this parameter. The distribution of properties by family can be seen in Figure 3.

Analogous to the first node of the tree, the third family was separated from the second node of the tree by the variable ∆HRC(c_1_) > 0.357. These variables measure the deviation of the value of the Harary-like index from the Coulomb matrix (HRC) for the expected values for all hydrogels measured under the same extrusion pressure (c_1_). With this index, global 3D representations of molecular structure and electrostatic interactions among atoms are quantified. Thus, the index is a measure of the branching and electrostatic interactions inside the repetitive units of the hydrogel [60]. This subset, representing 10.6% of the data, was considered a family, given its short path to the final classification nodes.

Finally, the third node level was analyzed, which creates the separation of the main families or clusters. In this case, the cut-off was defined as ∆HRG(c_3_) > 0.428. These are deviations of the HRG index of the hydrogel (chemical structure branching) repetitive unit, with respect to the average values for all hydrogels measured under the same condition c_3_ = nozzle. The third family emerged as the most complex in terms of the number of nodes on its path and was the family that represented the most points in the database, with 17 end nodes. (See Supplementary Materials File S1 for the IFPTML-DTC model structure). Other variables accounting for deviations ∆D_k_(c_j_) from structural parameters D_k_ of the hydrogel under study, with respect to the expected/average value for other hydrogels measured under different conditions, are used to define subfamilies inside this bigger family. The input variables more commonly used account for deviations ∆D_k_(c_j_) with respect to c_1_ = extrusion pressure, c_2_ = extrusion speed, and c_4_ = nozzle inner diameter. However, the deviations with respect to c6 = polymer temperature and c_7_ = syringe temperature do not enter the model (see Table 6).

The descriptors used by the model can be seen in Figure 3 and are described in Table 5. This table summarizes the number of times each descriptor appears in the model and the number of conditions to which it was linked to calculate the MA operator. It can be seen that the one that appears most often is the Wiener index, which refers to a topological index of the molecule, defined as the sum of the lengths of the shortest paths between all pairs of vertices of the chemical graph representing the non-hydrogen atoms in the molecule [61]. From this index, others have been used to define different ways of calculating the connections between atoms [62]. In the study by Bonchev et al., a mathematical scenario for analyzing the distances between atoms within polymers was analyzed. For this, Wiener indices were used, and the relationship of these indices with the branching and the lengths of the branches was shown. With this index and those defined later, the macroscopic properties of the polymers, in this case, hydrogels, can be analyzed qualitatively [63].

**Table 5:** Descriptors selected in the IFPTML-DTC model, with details of each, including abbreviation, full name, general description of the assessed characteristic, related condition or variable, and the frequency with which each descriptor was selected in the model.

Input Variables ∆D_k_(c_j_)	Descriptor Code	Name	Description	Related Condition (c_j_)	Condition Name	Nodes Count	Ref.
∆Wap_i_(c_4_)	Wap_i_	All-path Wiener index	Counts the number of bonds between pairs of atoms to generate a matrix. Does not take hydrogens into account.	4	Nozzle inner diameter	3	[64]
∆Wap_i_(c_1_)				1	Extrusion pressure	3	
∆WiDzv_i_(c_0_)	Wi_Dz(v)_i_	Wiener-like index from Barysz matrix weighted by van der Waals volume		0	Measured property	1	[61]
∆WiDzv_i_(c_9_)				9	Ethanol content	4	
∆WiCoulomb_i_(c_0_)	Wi_Coulomb_i_	Wiener-like index from Coulomb matrix		0	Measured property	2	[65]
∆WiCoulomb_i_(c_5_)				5	Layers printed	2	
∆HRG_i_(c_3_)	H_RG_i_	Harary-like index from reciprocal squared geometrical matrix	It counts the number of bonds of disordered atoms, always taking the shortest path.	3	Nozzle	2	[59,66]
∆HRG_i_(c_2_)				2	Extrusion speed	1	
∆HCoulomb_i_(c_1_)	H_Coulomb_i_	Harary-like index from Coulomb matrix		1	Extrusion pressure	3	
∆HCoulomb_i_(c_4_)				4	Nozzle inner diameter	1	
∆Mor01s_i_(c_3_)	Mor01s_i_	Moran autocorrelation of lag 1 weighted by I-state	It is a correlation of two signals between atoms close to each other in space.	3	Nozzle	1	[67]
∆GMTIV_i_(c_1_)	GMTIV_i_	Gutman molecular topological index by valence vertex degrees	A weighted sum that considers the vertices and valences of all pairs of atoms in a graph.	1	Extrusion pressure	2	[62,68]
∆GMTIV_i_(c_2_)				2		1	
∆SMTI_i_(c_1_)	SMTI_i_	Schultz molecular topological index		1		4	[48]
∆SMTI_i_(c_2_)				2		5	
∆IDMT_i_(c_0_)	IDMT_i_	Total information content on the distance magnitude		0	Measured property	2	
	f(v_i,j_)_ref_	Reference function	Value dependent on the property to be calculated.	-		2	

In this work, in addition to linear models, and for comparative purposes, three types of supervised learning-based neural networks were developed. The development of these models was carried out using TensorFlow [69] and Keras [70] libraries. Neural networks with varying degrees of complexity in terms of network type and the number of both hidden layers and neurons have been created. Similar to the previous models, the models that yielded the best results are available in the GitHub repository.

A multi-layer perceptron classifier (IFPTML-MLPC) is a type of ANN used in machine learning. It consists of multiple layers of dense, interconnected nodes, and each layer processes and transforms data using a set of weighted connections. The outcomes of these different-complexity IFPTML-MLPC neural networks can be observed in Table 6.

**Figure 4:**
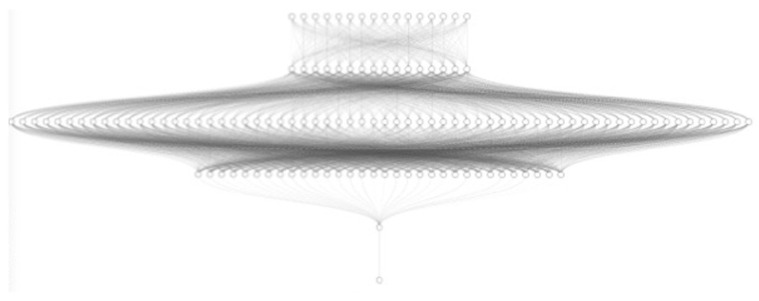
Architecture of the IFPTML-DEEP-ANN neural network with 16:16-64-32-32-32-1:1 configuration, showing the arrangement of layers and neurons in the model.

After testing an IFPTML-MLPC model in which the number of neurons and layers comprising the network has been varied, we observe that starting from the architecture of two hidden dense layers with 100 neurons each, the prediction performance does not significantly improve the results obtained with the IFPTML-DCT model. The results obtained with this type of neural network are somewhat better in terms of specificity, but there was no significant improvement. Therefore, a more complex deep learning ANN with the same architecture (16:16-64-32-32-1:1 ReLU activation, ADAM optimizer, binary cross entropy classification, Figure 4) was developed; however, in order to optimize the training, the number of epochs and batch size were varied. The neural networks defined as IFPTML-DEEP-ANN are neural networks with hidden dense layers. This means that in all the hidden layers comprising the neural network, every neuron is connected to all the preceding and subsequent neurons.

**Table 6:** Results of IFPTML-DEEP-ANN models trained with different levels of hidden neurons, showing observed versus predicted counts, sensitivity (Sn) and specificity (Sp) percentages, and area under the curve (AUROC) values.

Profile		Training				Validation		
	f(v_i,j_)	0 ^a^	1 ^a^	(%)	Par.	(%)	0 ^a^	1 ^a^
IFPTML-MLPC 1:1-100-100-1:1	0 ^b^	352	222	77.4	Sp	56.8	134	102
	1 ^b^	118	405	61.3	Sn	77.4	53	182
	AUROC	0.694				0.671		
IFPTML-MLPC 1:1-100-100-100-1:1	0 ^b^	491	83	85.5	Sp	78.8	186	50
	1 ^b^	170	353	67.5	Sn	63.8	85	750
	AUROC	0.765				0.713		
IFPTML-MLPC 1:1-100-1100-100-1:1	0 ^b^	187	82	85.7	Sp	79.2	187	49
	1 ^b^	171	352	67.3	Sn	63.0	87	148
	AUROC	0.765				0.711		

^a^ f(vi,j)pred = 0 or 1 predicted values, ^b^ f(vi,j)obs = 0 or 1 observed values.

Various models were tested regarding different epochs and batch sizes. As shown in Table 7, the results do not improve in the same way across different batch sizes, although an improvement in results was observed as the number of epochs increased. In the last IFPTML-DEEP-ANN case, in which 1000 epochs were tested, better results were obtained, even compared with the results of the previous linear models. As mentioned in other sections of this work, all these models are available in the web repository.

**Figure 5:**
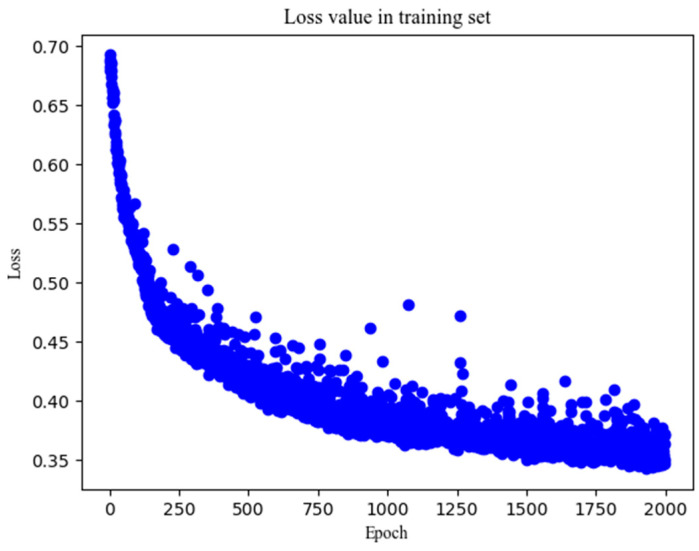
Evolution of the loss value as a function of the number of epochs during model training.

To perform a visual analysis of the quality parameters of the model developed using the IFPTML-DEEP-ANN technique, Figure 5 shows the change in the loss value as a function of the number of epochs that have been trained.

### 4.2. Experimental and Computational Case of Study of ChiMA Gel

This case study has two parts. The first is the experimental study of a set of bioprinted ChiMA scaffolds and the analysis of the taken image. Afterward, the results obtained with the predictive models will be shown.

#### 4.2.1. Experimental Characterization of Two New ChiMA and ChiMA + PEGDA Hydrogels

**Figure 6:**
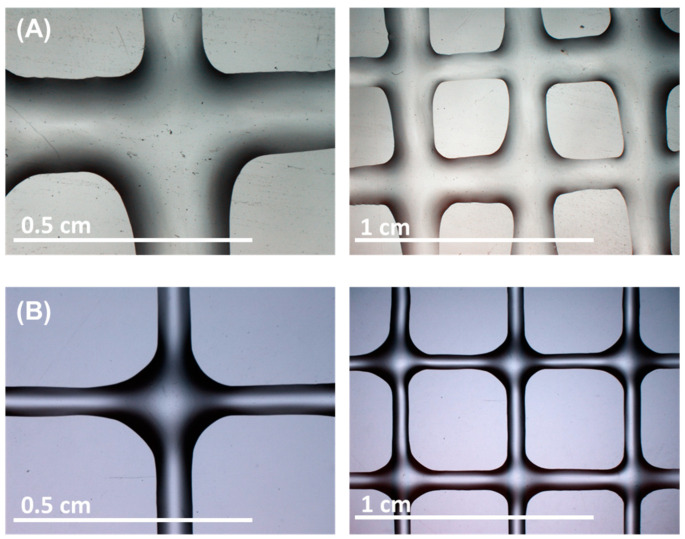
(**A**). ChiMA 3D-printed scaffold. (**B**). Three-dimensional-printed scaffold by ChiMA + PEGDA. Both images show the structures of the bioprinted scaffolds, highlighting the differences in the composition and morphology of the materials used.

Once a model was obtained to make the predictions (IFTPML-DTC), two hydrogel structures were printed to test the predictions made with the model and the actual results. The 3D printing of the ChiMA and ChiMA + PEGDA hydrogel inks was carried out as described in the corresponding section in the experimental part, obtaining the bioprinted designs shown in Figure 6. With the photographs of the inks of the ChiMA (Figure 6A) and ChiMA + PEGDA (Figure 6B), the width, length, perimeter, and area of the hydrogel grids were obtained with ImageJ software, as previously described. These data were used to calculate the printability parameters of uniformity and expansion shown in Table 8.

The results obtained from the image analysis and the model can be assumed to be as expected. The model was trained with a database in which most of the inputs are composed of a single hydrogel, which is why it was more accurate in predicting the results of those experiments that best fit the training data.

#### 4.2.2. IFPTML Computational Simulation of ChiMA Gel

In the previous sections, we described the synthesis and analysis study of this pure ChiMA hydrogel 3D bioprinting assays. Even so, there are several unmeasured properties and conditions that can be predicted using the model. In order to visualize the predictions made by the model in a simpler way, a heatmap has been generated, which can be seen in Table 9. In this table, the values of the two main bioprinting conditions are changed: c_1_ = extrusion pressure (kPa) and c_2_ = extrusion speed (mm/s). In Table 9, the probability of good (1) classification for each of the trials has been calculated while maintaining the rest of the conditions (c_3_ = 25 G, c_4_ = 254 μm, c_5_ = 1, c_6_ = 25 °C, c_7_ = 25 °C, c_8_ = 25 °C, c_9_ = N). To make the visualization of the values easier, the values from 0 to 1 have been colored with a red–orange–green color scale. In addition to the experimentally measured printing properties, the value of the integrity factor is predicted. The results can be seen in the heatmap in Table 8.

Although the results obtained in the experimental test and the predictions are not equal, they can be understood to be very close to reality. This may be because the margins for classification are very strict, and those impressions that, although below the limits, are close to the limits can be considered good results.

## 5. Conclusions

The generation of a database created from scratch based on bibliographic data on 3D printing with hydrogels constitutes the starting point of this work. This database comprises experimental measures of 16 properties associated with the printability of different hydrogels and includes more than 1200 tests. Subsequently, models with different complexities have been tested for predicting the quality of printed scaffolds. The developed nonlinear models showed better results than linear models and neural networks. In fact, the best of these models turned out to be the one based on lasing trees. Furthermore, the applicability of the methodology has been demonstrated by carrying out a study of a real case in our laboratories. This work offers valuable information on the factors that affect a 3D printing process. Thus, this contribution paves the way to minimizing the trial-and-error nature of the printing process by reducing the uncertainties inherent to it. Looking ahead, the database could be fed by us or other researchers, which would facilitate the development of models with even greater accuracy and efficiency.

## Figures and Tables

**Table 1:** List of conditions collected from the literature to describe bioprinting.

c_j_	Condition
c_1_	Extrusion pressure (kPa)
c_2_	Extrusion speed (mm/s)
c_3_	Nozzle
c_4_	Nozzle inner diameter (μm)
c_5_	Layers printed
c_6_	Mixture temperature (°C)
c_7_	Syringe temperature (°C),
c_8_	Platform temperature (°C)
c_9_	Ethanol content (Yes or No)

**Table 4:** Confusion matrices for the IFPTML-LDA and IFPTML-DTC models showing the observed and predicted case counts, along with the sensitivity (Sn) and specificity (Sp) percentages, for each model. The results are organized in the first column, allowing for a detailed comparison of the performance parameters between the two models.

Model	Data	Classes			f(vi,j)pred		
	Set	f(vi,j)obs	Stat.	(%)	nj	0	1
	training	0	Sp	72.8	558	406	103
IFPTML-LDA		1	Sn	80.9	539	152	436
	validation	0	Sp	71.0	252	179	50
		1	Sn	77.2	219	73	169
	Data	Classes			f(vi,j)pred		
	Set	f(vi,j)obs	Stat.	(%)	nj	0	1
IFPTML-DTC	training	0	Sp	88.4	562	497	74
		1	Sn	86.2	535	65	461
	validation	0	Sp	85.9	248	213	44
		1	Sn	80.3	223	35	179

**Table 7:** Results of IFPTML-DEEP-ANN models trained with the 16:16-64-32-32-32-1:1 architecture, varying the number of epochs and batch sizes. Includes performance metrics for each configuration.

Epoch	Batch Size	Train				Test				
		Sp (%)	Sn (%)	Ac (%)	AUC	Sp (%)	Sn (%)	Ac (%)	AUC	Loss
	32	66.9	80.1	73.2	0.832	61.8	82.6	72.2	0.783	0.611
100	64	84.8	66.7	76.2	0.807	78.8	63	70.9	0.761	0.582
	128	60.8	78.2	69.1	0.818	56.7	78.3	67.5	0.774	0.61
200	32	79.4	74.6	77.1	0.861	73.7	73.6	73.7	0.792	0.596
	64	69.8	83.4	76.3	0.858	64.0	78.3	71.1	0.788	0.614
	128	83.0	69.8	76.6	0.856	76.3	66.8	71.5	0.789	0.602
500	32	86.6	68.6	78.3	0.895	78.4	66	72.2	0.815	0.656
1000	32	83.5	80.7	82.1	0.906	75.0	78.7	76.8	0.830	0.659
2000	32	79.4	81.2	80.3	0.915	72.5	80.0	76.2	0.815	0.886

**Table 8:** Results obtained for ChiMA and ChiMA + PEGDA bioprint, including printing parameters, as well as observed and predicted classification using the model developed in this work.

	Parameter	Value	Classification Observed	Classification Predicted
ChiMA	Uniformity	0.94	1	1
	Expansion	6.44	0	0
	Porosity	0.38	0	0
ChiMA + PEGDA	Uniformity	0.98	1	0
	Expansion	2.78	0	0
	Porosity	0.69	0	0

**Table 9:** Prediction heatmap for ChiMA 1.5 *w*/*v*%, showing the probabilities of properties reaching desirable values when setting conditions and varying the values of c_1_ and c_2_.

		Extrusion Speed (mm/s) (c_2_)				Property
		1	7	10	25	
Extrusion P (kPa) (c_1_)	25	0.071	0.927	0.071	0.148	Expansion
	30	0.583	0.148	0.583	0.071	
	35	0.148	0.071	0.148	0.927	
	48	0.071	0.927	0.071	0.148	
	25	0.071	0.927	0.071	0.148	Pr
	30	0.583	0.148	0.583	0.071	
	35	0.148	0.071	0.148	0.927	
	48	0.071	0.927	0.071	0.148	
	25	0.927	0.148	0.927	0.071	U
	30	0.148	0.071	0.148	0.583	
	35	0.071	0.927	0.071	0.148	
	48	0.927	0.148	0.927	0.071	
	25	0.071	0.927	0.071	1.000	I
	30	0.583	1.000	0.583	0.071	
	35	1.000	0.071	1.000	0.927	
	48	0.071	0.927	0.071	1.000	
Color-scale for probability values		Low		Medium		High

## Data Availability

Lastly, this section includes a link to the article’s repository, where the initial database and all the code generated during this research are located, thus ensuring transparency and clarity. In addition, the source code of the new methods is available through a publicly accessible service and is distributed under an open-source license. https://github.com/hbediaga/hydrogel (accessed on 4 November 2024).

## Supplementary Materials

The following supporting information can be downloaded at: https://www.mdpi.com/article/10.3390/polym17010121/s1.
